# The complete chloroplast genome of queen’s crape-myrtle(*Lagerstroemia macrocarpa*)

**DOI:** 10.1080/23802359.2016.1176879

**Published:** 2016-06-20

**Authors:** Cuihua Gu, Luke R. Tembrock, Yunlong Li, Xiaoqing Lu, Zhiqiang Wu

**Affiliations:** a School of Landscape and Architecture, Zhejiang Agriculture and Forestry University, Hangzhou, People’s Republic of China;; b Department of Biology, Colorado State University, Fort Collins, CO, USA;; c Institute of Botany, Jiangsu Province and Chinese Academy of Sciences, Nanjing Botanical Garden Mem.Sun Yat-Sen, Nanjing, People’s Republic of China

**Keywords:** Chloroplast genome, *Lagerstroemia macrocarpa*, next-generation sequencing

## Abstract

The whole complete chloroplast genome of *Lagerstroemia macrocarpa* was assembled in this study. Total genome is 152,472 bp in length consisting of two inverted repeats of 17,562 bp separated by a large single-copy region and a small single-copy region of 84,050 bp and 33,295 bp, respectively. This genome contains 112 unique genes including 78 protein-coding genes, 4 ribosomal RNA genes and 30 transfer RNA genes. In 78 protein-coding genes, 8 genes (*atpF*, *ndhA*, *ndhB*, *petB*, *petD*, *rpl16*, *rpoC1*, *rps16*) contain one intron and three genes with two introns each (*clpP*, *rps12* and *ycf3*). This newly sequenced chloroplast genome supply highly variable information of polymorphisms within *Lagerstroemia* species.

*Lagerstroemia* (‘crape myrtle’) is the most economically genus in Lythraceae and comprises about 55 species (Koehne 1883; Furtado & Srisuko 1969; Qin & Graham 2007) which mainly distributed in tropical and sub-tropical habitats of southern China, Japan and northeast Australia (Egolf & Andrick 1978). Most *Lagerstroemia* species blossom from summer till fall with good features of colorful flowers, being easily propagated, strongly resistant to multiple pathogens (Wang et al. 2011). In terms of ornamental value of *Lagerstroemia*, more than 260 cultivars have been registered in the international authority of *Lagerstroemia* cultivars (http://www.usna.usda.gov/Research/Herbarium/Lagerstroemia/index.html). Due to the important value of *Lagerstroemia*, molecular tools have been employed to carry out identification of *Lagerstroemia* cultivars and interspecific hybrids (Pooler 2003; Pounders et al. 2007). *Lagerstroemia macrocarpa* (accession number: ZAFU 1507141) is deciduous tree with many-flowered terminal panicle which has valuable ornamental character and scattered in dry dipterocarp forest and open forests in Burma (Myanmar) and Laos (Khammouan). We got sample and specimens from Xishuangbanna Tropical Botanic Garden (XTBG) where *L. macrocarpa* has amount of introduction.

In contrast to large nuclear genomes, the chloroplast genome has a highly conserved circular DNA structure varying from 115 to 165 kb with uniparental inheritance, low recombination rates, conserved gene order and sequence similarity across the land plants (Palmer 1985; Ravi et al. 2008; Wicke et al. 2011). Plant chloroplast genomes have been a valuable source of informative markers in phylogenetic studies (Moore et al. 2010; Wang et al. 2010; Wu & Ge 2012), plant barcoding (Day & Goldschmidt-Clermont 2011) and biogeographical researches among populations (Wang et al. 2011). With the decreasing cost of next-generation sequencing approaches, sequencing whole chloroplast genomes is becoming more popular (Soltis et al. 2013). To date, more than 800 land plant species’ completed chloroplast genomes can be searched through the National Center for Biotechnology Information (NCBI) public database (Wu et al. 2015).

The raw Illumina reads were trimmed and filtered by quality score using Trimmomatic v0.3 (Bolger et al. 2014) with the following settings: leading: 3, trailing: 3, sliding window: 4:15 and minlen: 50. Then the CLC Genomics Workbench v7 (CLCbio) (http://www.clcbio.com) was employed to process *de novo* assembly of reads from *L. macrocarpa* with the default parameters. Three separate *de novo* assemblies (PE reads, single-end forward reads and single-end reverse reads) were made (Wu et al. 2015) and then combined into a single assembly. The whole chloroplast genome for *L. macrocarpa* was finished at 152,472 bp in length after combining the Sanger and Illumina sequence data. Through mapping the paired reads onto the finished genome, we verified our assembled length for the finished chloroplast genome with 1,473,293 (5% of the total reads) mapped reads across the whole chloroplast genome with at least 950 reads per position.

The final chloroplast genome was annotated with DOGMA (http://dogma.ccbb.utexas.edu/) and manually fixed exon-intron junctions (Wu et al. 2015). The complete chloroplast genome sequence was submitted to GenBank under the accession number of KU821692. Seventy shared protein coding genes were extracted and used to constructed the NJ tree with 500 bootstrap replicates by MEGA7 (www.megasoftware.net) (Figure 1) followed by the method used in (Wu et al. 2015).

**Figure 1.**
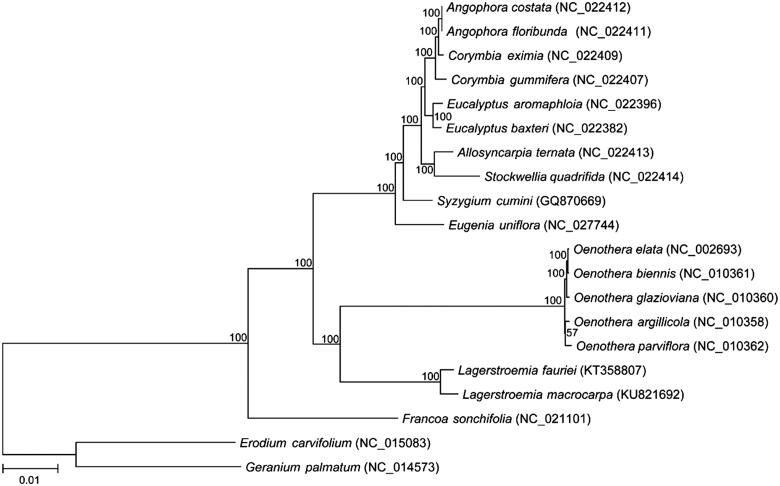
The phylogenetic relationships among 18 species with two other species from Geraniaceae family as outgroup were constructed by neighbour-joining (NJ) method using MEGA7. The numbers on the branches are bootstrap values. GenBank accession numbers are listed following each species.

The *L. macrocarpa* chloroplast genome is 152,472 bp in size with 37.6% GC content, consists of a pair of inverted repeats of 17,562 bp, separated by a large single-copy region and a small single-copy region of 84,050 bp and 33,298 bp, respectively. 112 unique genes were annotated including 78 protein-coding genes, 4 ribosomal RNA genes and 30 transfer RNA genes. Among these protein-coding genes, eight genes (*atpF*, *ndhA*, *ndhB*, *petB*, *petD*, *rpl16*, *rpoC1*, *rps16*), six tRNA genes with one intron each (*trnA^GUC^*, *trnG^UCC^*, *trnI^GAU^*, *trnK^UUU^*, *trnL^UAA^*, *trnV^UAC^*) and three genes (*clpP*, *rps12* and *ycf3*) have two introns.
